# Vector-borne transmission of Zika virus in Europe, southern France, August 2019

**DOI:** 10.2807/1560-7917.ES.2019.24.45.1900655

**Published:** 2019-11-07

**Authors:** Sandra Giron, Florian Franke, Anne Decoppet, Bernard Cadiou, Thierry Travaglini, Laurence Thirion, Guillaume Durand, Charles Jeannin, Grégory L’Ambert, Gilda Grard, Harold Noël, Nelly Fournet, Michelle Auzet-Caillaud, Christine Zandotti, Samer Aboukaïs, Pascal Chaud, Saby Guedj, Lakri Hamouda, Xavier Naudot, Anne Ovize, Clément Lazarus, Henriette de Valk, Marie-Claire Paty, Isabelle Leparc-Goffart

**Affiliations:** 1 Santé publique France (French National Public Health Agency), Marseille, France; 2 Regional Health Agency of Provence-Alpes-Côtes d’Azur (ARS Paca), Marseille, France; 3 Entente interdépartementale pour la démoustication du littoral méditerranéen (EID Méditerranée), Montpellier, France; 4 Unité des Virus Emergents (UVE: Aix-Marseille Univ – IRD 190 – Inserm 1207 – IHU Méditerranée Infection), Marseille, France; 5 Institut de Recherche Biomédicale des Armées, National Reference Laboratory for Arboviruses, Marseille, France; 6 Santé publique France (French National Public Health Agency), Saint-Maurice, France; 7 Médecin généraliste, Hyères, France; 8 Eurofins Biomnis, Lyon, France; 9 Public Health Emergency Operations Centre, Division of Surveillance and Health Security, Ministry of Health, General Directorate for Health, Health Emergencies Crisis Management Centre, Paris, France

**Keywords:** Zika virus, vector-borne transmission, France, *Aedes albopictus*

## Abstract

On 1 October 2019, a locally-acquired Zika virus disease case was laboratory confirmed in Hyères, Var department. Active case finding identified two additional locally-acquired cases living within 90 m, with symptom onset 8 days before the index case. Extensive patient interviews did not yield information supporting transmission through sexual contact or substances of human origin. Vector-borne transmission by local *Aedes albopictus* mosquitoes is the most likely mode of transmission. Here we describe the public health response.

Zika virus (ZIKV) is an arbovirus that caused a pandemic in the Pacific and the Americas from 2013 to 2016, which led to the World Health Organization (WHO) declaring it a public health emergency of international concern in February 2016, and to the identification of a causal link between ZIKV and birth defects [1]. However, no vector-borne transmission was documented in Europe. Here we report the investigations and control measures implemented in response to the detection of three cases of ZIKV disease with symptom onset within 8 days in patients all residing in the same neighbourhood in the municipality of Hyères, Var department, southern France.

## Epidemiological and laboratory investigations

Zika, chikungunya and dengue virus infections are mandatorily notifiable in France. Every year at the start of the season of vector activity, from May through November, health professionals are reminded of the importance of testing patients with symptoms compatible with one these diseases for all three viruses and of notifying all confirmed cases.

Early October 2019, the French National Reference Centre (NRC) for Arboviruses in Marseille confirmed a case of recent ZIKV infection on the basis of detecting ZIKV RNA in a blood specimen from day 2 of symptom onset by using transcription-mediated amplification (TMA) technology-based Aptima Zika Virus assay (Hologic, Marlborough, Massachusetts, United States), and detecting IgM and ZIKV-specific neutralising antibodies in a blood sample collected 12 days after symptom onset. The otherwise healthy patient was in their 50s and had developed febrile rash eruption and asthenia around 15 August. The patient and their spouse did not report any history of recent travel to a Zika-endemic country, transfusion of blood or blood products, or organ transplantation. Investigations were initiated in order to explore the possibility of local vector-borne transmission to identify any additional cases and to determine the affected area.

## Active case finding

The regional health authority of Provence-Alpes-Côtes d’Azur and Santé publique France, the French National Public Health Agency, carried out active case-finding by contacting all general practitioners, physicians, medical laboratories and emergency services in the municipality. We requested them to test and report to the regional health authorities, any patient with symptoms compatible with ZIKV infection (i.e. rash, fever, conjunctivitis) retrospectively and prospectively, and independent of travel history. In addition, we conducted a door-to-door case-finding campaign on 10 October, visiting all households within a 100 m radius of the index case’s home to identify and test any household member or visitor with symptoms compatible with ZIKV infection, and to identify persons with a recent history of travel to a ZIKV-endemic area.

## Case definition

For this investigation a case was defined as a person presenting since 1 July 2019 with a rash, with or without fever and at least one of the following signs and symptoms: arthralgia, myalgia or conjunctivitis/hyperaemia. Laboratory confirmation was based on the detection of ZIKV nucleic acid from a clinical specimen, detection of ZIKV-specific IgM antibodies in a serum sample and confirmation by neutralisation test or seroconversion or fourfold increase in the titre of ZIKV-specific antibodies in paired serum samples. A probable case was defined based on detection of ZIKV-specific IgM antibodies in a serum sample alone.

Households within a 200 m radius all received a letter of information and the recommendation to consult their general practitioner in case of symptoms suggestive of ZIKV infection since 1 July 2019 [2]. This window period of 45 days prior to disease onset of the index case was chosen because it corresponds to the delay between two cases belonging to the same transmission cycle. In parallel, we reviewed the French arbovirus diseases surveillance database to look for a possible primary case among the imported ZIKV cases.

The door-to-door case finding campaign allowed us to investigate 27 of 55 households identified within the 100 m radius around the home of the index case, and led to the identification of two additional probable cases who had developed fever and rash 8 days before the onset of the rash in the index case. The two cases, otherwise healthy middle-aged adults who lived within 90 m from the index case. The NRC confirmed the presence of ZIKV-specific IgM and anti-flavivirus IgG antibodies using an in-house ELISA with precipitated and inactivated virus as antigen in samples taken for both cases 2 months after symptom onset. A neutralisation test is ongoing and nucleic acid testing on samples of urine and whole blood is planned for one of the cases. A semen sample tested negative in RT-PCR. Public health authorities are currently looking for biological samples from the three cases that may have been collected shortly after disease onset, which may still be available in medical laboratories and from which viral RNA could be obtained. Both cases and their partners did not report travel to a ZIKV-endemic area. The extensive interviews with the two cases did not yield any information supporting transmission through sexual contact or substances of human origin (SoHO).

Review of the national arbovirus database did not identify any viraemic imported ZIKV case in Var department. The door-to-door campaign did not identify any persons with recent travel to a ZIKV-endemic area.

A hotel was identified within the affected area. The hotel sent a letter written by the regional health agency to all guests that stayed there between 1 July and 1 October 2019, informing them of possible ZIKV circulation in the area and advising them to consult a healthcare professional in case of pregnancy, irrespective of the presence of symptoms. Guests were also asked to contact the regional authority if they had travelled to a ZIKV-endemic area within 15 days before their hotel stay.

## Entomological investigations and vector control measures

The Entente Interdépartementale pour la Démoustication du littoral Méditerranéen (EID Méditerranée), the public mosquito control operator, carried out investigations inside and around the three cases’ homes as well as places visited by the cases during their viraemia.

The affected area is a residential area with gardens and private swimming pools, and many second homes that were not occupied at the time of the survey. Residents present reported mosquito nuisance. Adult mosquito traps (BG-Sentinel, Biogents, Regensburg, Germany) and ovitraps were placed in several gardens to estimate vector densities and for further vector competence studies. Presence of the virus in the saliva of captured mosquitoes was also tested using Whatman FTA technology. Between 9 and 18 October, the vector control operators implemented vector control measures around the three cases’ homes and in the area. *Bacillus thuringiensis* serovar *israelensis* H14 (Bti) was used for larvae and deltamethrin for adult mosquitoes. Larval breeding sites such as emptied swimming pools, little containers, etc. were treated with Bti or removed (e.g. spilled) when possible.

## Pregnant women and substances of human origin

ZIKV infection during pregnancy has been shown to increase the risk of congenital malformations, birth defects, preterm birth and miscarriage [1,3,4]. Maternal-foetal transmission of ZIKV may occur in all trimesters of pregnancy and vertical transmission has been estimated to occur in 26% of foetuses of ZIKV infected mothers in French Guiana [4].

The regional health authorities are in the process of identifying any pregnant women in the affected area and surroundings through the national health insurance scheme (Caisse Nationale d’Assurance Maladie) in order to inform them of the possibility of virus circulation over the last months, the need to protect themselves against mosquito bites and the need to consult their physician or midwife. Health professionals involved in antenatal care, obstetrics and medically-assisted reproduction are currently being informed and reminded of the existing guidelines for the follow-up of pregnant women potentially exposed to ZIKV and of precautions for women and couples planning a medically assisted pregnancy.

ZIKV can also be transmitted through transfusion of blood and blood products, through organ transplantation and by sexual contact [5,6]. The working group for the safety of SoHO of the High Council of Public Health (Haut Conseil de la santé publique) is preparing a risk assessment and will decide if further measures to secure the safety of SoHO are needed.

## Discussion

Our findings are strongly suggestive of the occurrence of three, vector-borne, autochthonous cases of ZIKV infection in the Var department in France. Their occurrence over a period of 8 days and within a 90 m distance suggests they belong to a same transmission cycle. *Aedes albopictus* has been established in Hyères since 2009 and is known to be abundant in the region, where it is normally active from May through November [7]. Vector control measures were put in place rapidly after detection and an awareness campaign was carried out to inform health professionals about the possible risk of infection, particularly in pregnant women. We wish here to inform about public health measures and raise further awareness on the risk of ZIKV infection in south of France in summer 2019.

ZIKV is a positive-sense RNA flavivirus in the family Flaviviridae [1]. Most infections are either asymptomatic or cause a mild illness with fever, rash, conjunctivitis, muscle and joint pain, or headache [8]. However, neurological conditions, such as Guillain–Barré syndrome, have been reported to occur at the frequency of 2 to 3 cases in every 10,000 infected adults and children [9]. *Aedes aegypti* is the major mosquito vector for ZIKV transmission. However, the competence of European populations of *Ae. albopictus* to transmit ZIKV has been shown in laboratory studies [10-12]; the degree of vector competence in laboratory studies varies considerably with the origin of the ZIKV and the origin of the *Ae. albopictus* colony [10]. *Ae. albopictus* was implicated in the 2007 ZIKV outbreak in Gabon [13] and possibly in Mexico in 2016 [14]. There is, however, no evidence of *Ae. albopictus* being implicated in ZIKV transmission in Europe thus far. During the massive outbreak of the Asian lineage of ZIKV in the Americas in 2016, no autochthonous vector-borne case was detected in European Union/European Economic Area (EU/EAA) countries, despite of the importation of 584 cases between May and October 2016, among residents of areas with established presence of *Ae. albopictus* [15].

Because our investigations took place more than 45 days after the onset of symptoms of the last case, viral RNA could not be obtained from patients’ specimens or captured mosquitos. Hence we have no phylogenetic information on the ZIKV that infected the three cases although the search for a sample collected early after symptom onset is still ongoing. We could not identify a potential primary imported case and its geographical origin of infection.

This observation of a vector-borne ZIKV transmission by *Ae. albopictus* in southern France is of importance considering the widespread establishment and abundance of *Ae. albopictus* in large areas of southern Europe, and the continuous importation of ZIKV by infected travellers returning from areas with ZIKV transmission. Although transmission of ZIKV has declined in the Americas since 2016, ZIKV infections still continue to occur in the Americas and other regions [16].

The likelihood that vector-borne transmission in Hyères is still ongoing is very low as the environmental conditions are currently not favourable for a sustained transmission [17]. However, we cannot exclude that some people who travelled to Hyères this summer may have become infected. To address this, we alerted and raised awareness among other national public health institutes on this event through the Early Warning and Response System of the European Union and the WHO’s Disease Outbreak News [18]. The event was also widely covered by the media in France and this may have contributed to raising awareness among the general public. To date, no infection in people who visited the area over the summer has been notified to us.

Further investigations to document this event and to assess if and how ZIKV can establish a sustainable transmission cycle involving *Ae. albopictus* in southern Europe are ongoing. Also in progress are efforts to obtain viral RNA, and isolate the virus from the cases and captured mosquitoes. Local *Ae. albopictus* mosquitoes are being captured for vector competence studies. A seroprevalence study of ZIKV-specific antibodies will be carried out in the coming weeks to further determine the extent of viral transmission and capture any asymptomatic cases not identified by the active case finding.
